# Genus *Parkia*: Phytochemical, Medicinal Uses, and Pharmacological Properties

**DOI:** 10.3390/ijms22020618

**Published:** 2021-01-09

**Authors:** Mohammed S. M. Saleh, Juriyati Jalil, Satirah Zainalabidin, Ahmad Yusof Asmadi, Nor Hidayah Mustafa, Yusof Kamisah

**Affiliations:** 1 Department of Pharmacology, Faculty of Medicine, Universiti Kebangsaan Malaysia, Cheras, Kuala Lumpur 56000, Malaysia; medsaleh@ukm.edu.my; 2 Drug and Herbal Research Centre, Faculty of Pharmacy, Universiti Kebangsaan Malaysia, Kuala Lumpur 50300, Malaysia; juriyatijalil@ukm.edu.my (J.J.); norhidayahmustafa91@gmail.com (N.H.M.); 3 Program of Biomedical Science, Centre of Toxicology and Health Risk Study, Faculty of Health Sciences, Universiti Kebangsaan Malaysia, Kuala Lumpur 50300, Malaysia; satirah@ukm.edu.my; 4 Unit of Pharmacology, Faculty of Medicine and Defence Health, Universiti Pertahanan Nasional Malaysia, Kem Sungai Besi, Kuala Lumpur 57000, Malaysia; draayusof@gmail.com

**Keywords:** *Parkia*, *Mimosoideae*, traditional medicine, secondary metabolite, pharmacological activities

## Abstract

The genus *Parkia* (Fabaceae, Subfamily, Mimosoideae) comprises about 34 species of mostly evergreen trees widely distributed across neotropics, Asia, and Africa. This review aims to provide an overview of the current status of the species from the genus *Parkia* in terms of its relationship between its phytochemistry and medical uses. Comprehensive information on *Parkia* species was retrieved from electronic databases, which were Web of Science, ScienceDirect, PubMed, and Google Scholar. This review identified nine species from genus *Parkia* with properties of medicinal use. They are used traditionally to treat several ailments, such as diabetes, diarrhea, wounds, hypertension, cough, chronic piles, conjunctivitis, and measles. The most common species studied are *P. biglobosa*, *P. speciosa*, *P. javanica*, *P. bicolor*, *P. biglandulosa*, *P. filicoidea*, and *P. clappertoniana*. A considerable number of secondary metabolites, such as terpenoids, phenolic acids, flavonoids (aglycone and glycosides), and numerous volatile compounds have been identified in this genus, which are responsible for their diverse pharmacological activities. Their extracts, pure compounds and seed lectins have been reported for their anticancer, antimicrobial, antihypertensive, antiulcer, antidiabetic, anti-inflammatory, antioxidant, antimalarial, hepatoprotective, and antidiarrheal activities. The information gathered in this review might be of help for future studies in terms of the current knowledge on the link between the phytochemical components and medicinal uses. This could facilitate more discoveries on its potentials particularly in the pharmacological characteristics and potential to be developed into modern medicines.

## 1. Introduction

*Parkia* is a genus of flowering plants belonging to the family Fabaceae (subfamily, Mimosoideae) with pan-tropical distribution [1]. The word *Parkia* was named after the Scottish explorer Mungo Park, who drowned in the Niger River, Nigeria in January 1805 [2]. Thirty-one species from this genus were reported in 1995 [3]. Another four more species were discovered in 2009 [4]. Out of these species, 10 species found in Asia, four in Africa, and 20 in neotropics. Meanwhile, according to a plant list (2018), 80 scientific names are recorded from the genus *Parkia* containing 41 accepted names and 39 synonym species (The Plant List, 2018). These plants bear fruits called pods. Each pod contains up to 25–30 seeds. Many species from *Parkia* have been reported to be rich in carbohydrate [5,6,7], protein [8,9,10] and minerals [11,12,13,14].

From our extensive research regarding the biological, pharmacological, and phytochemical constituents of species from the genus *Parkia*, only 12 species have been scientifically investigated so far. Out of these, nine were studied on phytochemical analysis and determination of biological activities, which include *P. bicolor*, *P. biglobosa*, *P. biglandulosa*, *P. filicoidea*, *P. clappertoniana*, *P. javanica* (synonyms: *P. roxburghii*, *P. timoriana*), *P. pendula*, *P. platycephala*, and *P. speciosa*. The most frequently investigated over a wide range of ailments are *P. biglobosa* and *P. speciosa*. Studies on the remaining three species (*P. velutina*, *P. nitida*, and *P. polyads*) focused on morphological variations and environmental distribution. This review aims to collate the present state of medicinal uses and phytochemistry of the genus *Parkia* for future studies. The link between the phytochemical components and medicinal uses of the current knowledge is discussed with the hope it would expedite more discoveries that have potentials to be developed into modern medicines.

## 2. Methodology

**Figure 1:**
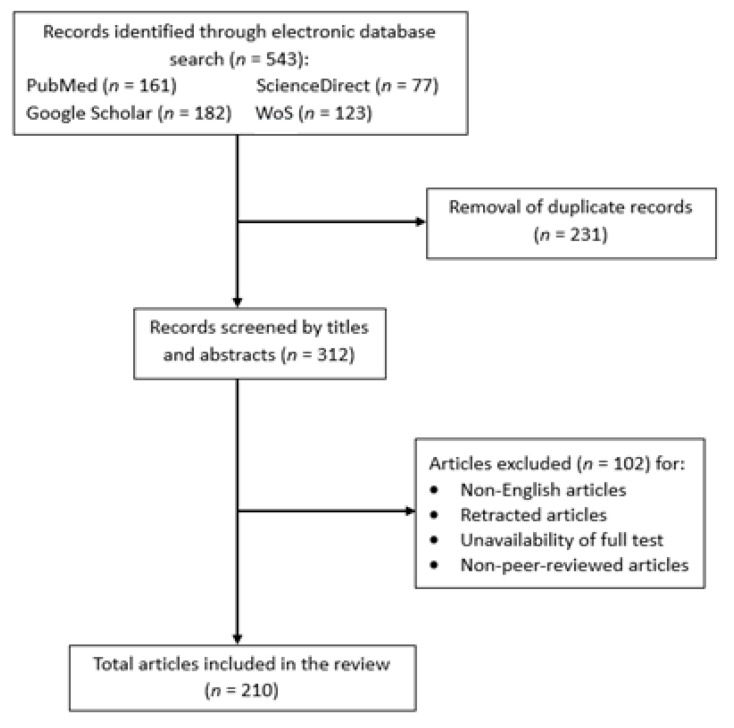
The preferred reporting items for systematic review and meta-analysis flowchart indicating the numbers of identified, screened, included and excluded articles in the review.

Information of the ethnobotanical use of plants from genus *Parkia* was retrieved from electronic databases which were ScienceDirect, Web of Science, Google Scholar, and PubMed, using search terms of “*Parkia* AND ethnobotanical”, *Parkia* AND pharmacological”, “*Parkia* AND pharmaceutical”, “*Parkia* AND toxicological studies”, “*Parkia* AND bioactivity”, “*Parkia* AND phytochemistry”, “*Parkia* AND ethnomedicinal”, and “*Parkia* AND morphological”. A total of 543 abstracts of research articles books, and conference papers published from 1961 to 2020 were obtained. Duplicates (*n* = 231) were removed. From the remaining 312 records, one hundred and two titles were excluded due to unavailability of full text or not published in English. Only articles and abstracts published in English were included. Two hundred and ten research articles and book chapters containing relevant and useful information were included in this review (Figure 1). Information gathered on the traditional uses, pharmacology and bioactive compounds identified from *Parkia* genus were summarized in the form of two tables and four figures. Chemical structures of bioactive compounds reported were drawn using ChemDraw software 16.0 (PerkinElmer Informatics, Waltham, MA, USA).

## 3. Traditional Medicinal Uses

*Parkia* species are being used across all tropical countries to cure different ailments. Virtually, all parts of *Parkia* plants are utilized traditionally for different medicinal purposes. The materials of different parts of *Parkia* plants are processed as paste, decoction, and juice for the treatment of various ailments (Table 1). Almost all reported *Parkia* species are used in different forms to cure diarrhea and dysentery [15]. Different parts of *P. biglobosa*, *P. clappertoniana*, *P. roxburghii*, and *P. speciosa* are reported to be traditionally used for the treatment of diabetes [16,17,18]. Furthermore, skin-related diseases, such as eczema, skin ulcers, measles, leprosy, wound, dermatitis, chickenpox, scabies, and ringworm are treated using leaves, pods, and roots of *P. speciosa* and *P. timoriana* [19,20,21]. The stem barks of *P. bicolor*, *P. clappertoniana*, *P. biglobosa*, *P. roxburghii* as well as roots of *P. speciosa* are applied in the form of paste and decoction to treat different skin problems [22,23,24,25]. Decoction and paste of stem bark, pod, or root of *P. biglobosa* and *P. speciosa* are used to treat hypertension [22,26,27]. Moreover, stem barks of *P. bicolor*, *P. biglobosa* and leaves of *P. speciosa* are used for severe cough and bronchitis [28,29,30]. These aforementioned uses suggested that *Parkia* plants are likely to contain constituents with broad and diverse biological activities, such as antidiabetic, antimicrobial, antihypertensive, and anti-inflammatory.

## 4. Phytochemistry of Genus *Parkia*

Among the numerous species of *Parkia* plant, the chemistry of only few are known. However, different parts of the reported ones have been validated as good sources of phenolic compounds [11,31,32], saponins [33,34,35], terpenoids [35,36,37], steroids [23,38,39], tannins [38,39,40], fatty acids [23,41], and glycosides [42,43,44].

Various phytochemicals are found in the stem barks, leaves, seeds, and pods of these plants. The stem bark of *P. biglobosa* is reported to contain phenols, flavonoids, sugars, tannins, terpenoids, steroids, saponins [11,38], alkaloid, and glycosides [35,43,45], while the leaves contain glycosides, tannins, and alkaloids in trace amount [11,23,46], in addition to flavonoids, phenols, and anthraquinones [47]. Phytochemical screening of the seeds shows the presence of saponins, alkaloids, flavonoids, polyphenols, terpenoids, glycosides and tannins [48,49]. Fermentation or roasting of *P. biglobosa* seeds results in the alteration of the bioactive components.

*P. bicolor* leaves contain chemical constituents similar to that of *P. biglobosa* such as glycosides, tannin, and alkaloids in trace amount [23]. The stem bark of *P. bicolor* contains alkaloids, tannins, saponins, glycosides, flavonoids, and terpenoids [35], while *P. biglandulosa* contains tannins, saponins, and glycosides, and *P. filicoidea* possesses flavonoids, sugars, saponins, and tannins [50]. The seed of *P. javanica* contains flavonoid, saponins, alkaloids, terpenoids, anthraquinones, steroids, and glycosides [44]. The pods are reported to have tannins, flavonoids, and saponins, all of which are significantly diminished when subjected to various processing methods, such as ordinary and pressure cooking methods [51,52]. Alkaloids, glycosides, saponins, and tannins are present in the whole plant of *P. clappertoniana* [31]. Phytochemical analysis of the leaves of *P. platycephala* revealed the presence of phenols, terpenoids, flavonoids [53], tannins and saponins [54]. Furthermore, flavonoids, alkaloids, phenols, and terpenoids were reported to be present in all parts of *P. speciosa* plant [37].

Phytochemicals (primary and secondary metabolites) are well known for their vast medicinal benefits to plants and human [100]. The primary metabolites—such as carbohydrate, proteins, chlorophyll, lipids, nucleic, and amino acids [101,102,103]—are responsible for plants’ biochemical reactions such as respiration and photosynthesis [102]. The secondary metabolites are majorly alkaloids, phenols, terpenoids, flavonoids, saponins, steroids, tannins, and glycosides, which play important roles in protecting the plants against damages and improving plant aroma, coloration and flavor [101,103], The phytochemicals are present in various parts of the plants especially in the three major parts viz. the leaves, stems and roots. Their percentage composition in each plant may vary depending on environmental conditions, variety and processing methods [101]. Previous studies have shown that phenolic compounds are the most abundant and widely distributed phytoconstituents (45%), followed by steroids and terpenoids (27%), and alkaloids (18%) [101,104]. Alkaloids, flavonoids, tannins, and phenolic compounds are the most common constituents that have been studied in phytochemistry [104,105]. Several compounds from these classes have been identified and investigated from *Parkia* plants for various pharmacological activities. Despite the enormous reports on the phytochemical screening of different species from the genus *Parkia*, structure identification and purification of compounds from these species are scarcely reported compared to other genera. The compounds were identified using high-performance liquid chromatography with diode-array detector (HPLC-DAD), liquid chromatography mass spectrometry (LCMS), flow analysis-ionization electrospray ion trap tandem mass spectrometry (FIA-ESI-IT-MS), gas chromatography time-of-flight mass spectrometry (GC/ToF-MS), high-performance liquid chromatography-electrospray ion mass spectrometry (HPLC-ESI-MS), and chromatographic purification from the fraction and characterization through nuclear magnetic resonance (NMR).

### 4.1. Polyphenolic Compounds

**Figure 2:**
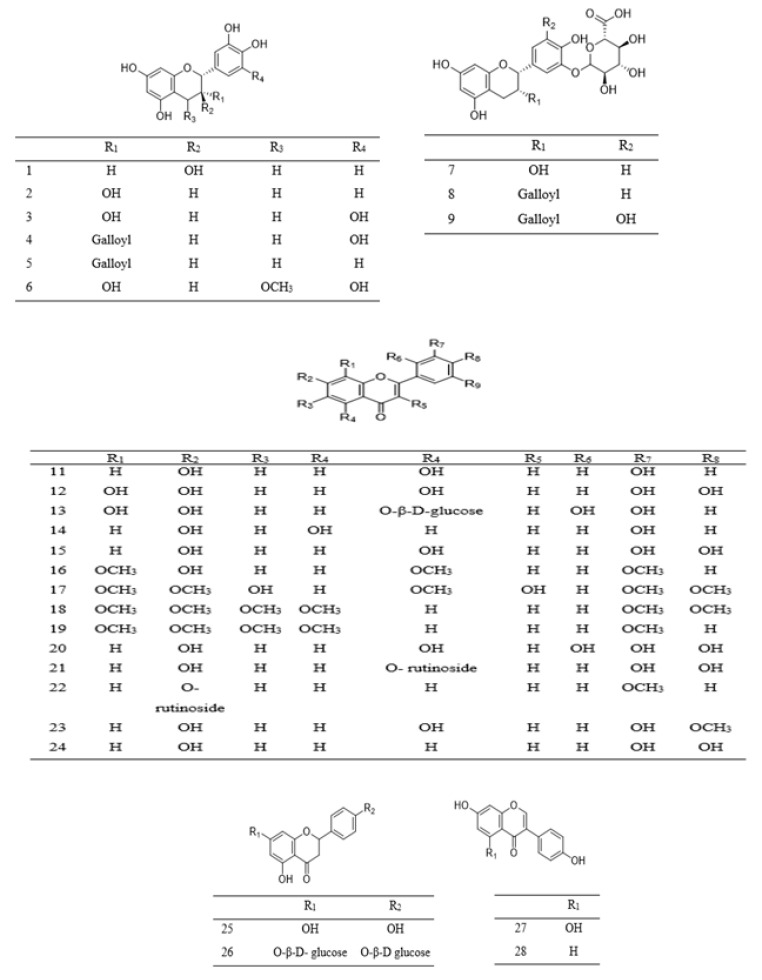
Structural formulas of polyphenolics **1**–**28**, as previously listed in Table 2.

**Figure 3:**
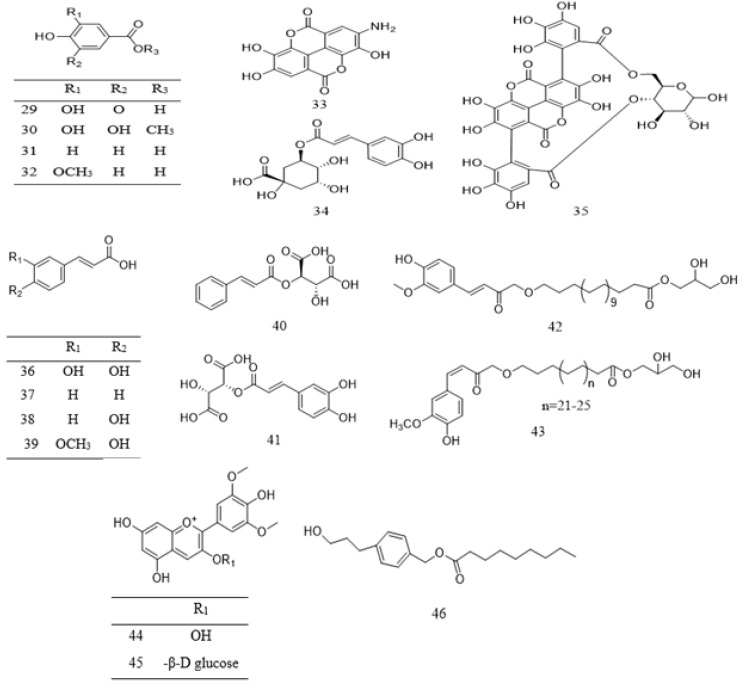
Structural formulas of polyphenolics **29**–**46**, as previously listed in Table 2.

Phenolic compounds found in *Parkia* species are grouped into simple phenol (**10** and **31**), phenolic acids **29**–**41**, flavone **15**–**19** and **24**, flavanone **25**–**26**, flavonol **11**–**14** and **20**–**22**, methoxyflavonol **23**, as well as flavanol **1**–**10** (Table 2). Phenolic acids are mostly found in the pods and edible parts of *Parkia*, while polyphenolic compounds are present in the leaves, stem barks, roots, or seeds. The most commonly reported flavonoid in *Parkia* species are flavanol **1** and its isomer **8**, which are obtained from the pod and bark of *P. speciosa* and *P. biglobosa*, respectively [106,107,108] and the remaining flavanols **11**–**18** are mainly galloylated catechins. Compound **11** is isolated from ethyl acetate fraction of *P. roxburghii* pod [18], while compounds **12**–**18** are identified from the ethyl acetate fraction of root/stem of *P. biglobosa* [18]. One methoxyflavonol **23**, two flavanone **26**–**27** and isoflavones **27**–**28** are identified in the edible parts of *P. javanica* [108]. A new flavanone, naringenin-1-4′-di-*O*-ß-D-glucopyranoside **26** is isolated from *n*–butanol fraction of *P. biglobosa* [109], while a new phenylpropanoid is elucidated as 4-(3-hydroxypropyl)benzyl nonanoate from the leaves of *P. javanica* [110]. Isolation of compounds **42**–**43** for the first time as a pure compound was reported from the ethanol extract of *P. biglobosa* bark [111]. The structures of these compounds are illustrated in Figure 2 and Figure 3.

### 4.2. Terpenoid and Steroid

**Figure 4:**
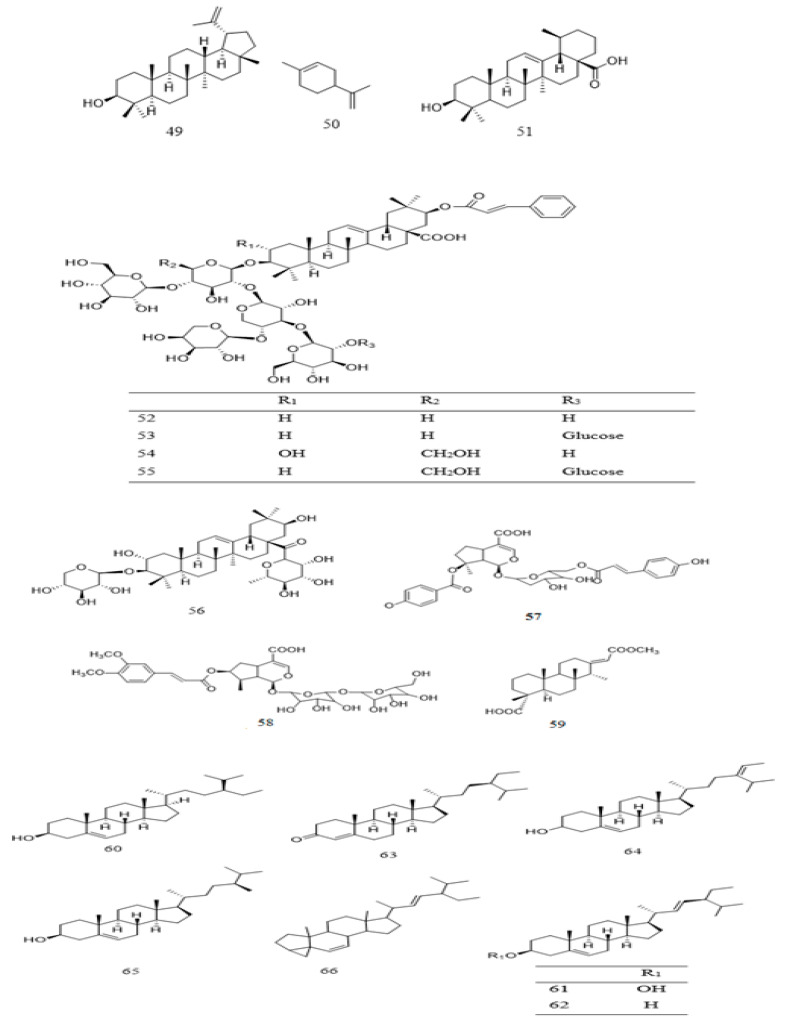
Structural formulas of terpenoids **49**–**59** and steroids **60**–**66**, as previously listed in Table 2.

To date, few terpenoid compounds have been reported in *Parkia* plants. Most of these compounds were identified from barks, roots, leaves, and seeds of *Parkia* plants. One is monoterpenoid **50** with two of its glucosides **57** and **58**, a diterpene **49**, while the rest are triterpenoid **49** and **51**–**56** (Table 2 and Figure 4). Seven out of the triterpenoids **52**–**58** were reported as new compounds. Only **49** is reported in three species (*P. biglobosa*, *P. bicolor*, and *P. speciosa*). Two of the new compounds **57** and **58** are iridoid type of terpenoidal glycoside purified from methanol extract of *P. javanica*, together with ursolic acid and other steroidal compounds [42]. Compounds **52**–**56** are isolated through different chromatographic techniques from 80% methanol extract of *P. bicolor* root, with a known diterpene **59** and a benzene glucoside **105**. These compounds are reported to exhibit moderate antiproliferative activity with median inhibitory concentration (IC_50_) ranging from 48.89 ± 0.16 to 81.66 ± 0.17 µM [118].

Steroidal compounds are also reported in the genus of *Parkia* (Table 2 and Figure 4). β-Sitosterol (**60**) is one of the major components in *P. speciosa* [120] and *P. biglobosa* seeds [121]. The steroid together with stigmasterol are purified from recrystallization of chloroform/methanol fraction of *P. speciosa* seeds. Its composition in *P. biglobosa* seeds was reported to be about 377 mg/100 g dry weight [122]. It is also purified from methanol extract of *P. javanica* leaves [42]. Apart from **60**, **61**, and **65**, which are present in *P. javanica* and/or *P. biglobosa*, all other steroids **62**–**64** and **66** reported from different studies are found in *P. speciosa* seeds. Other than β-sitosterol (**60**), stigmasterol (**61**), and campesterol (**65**) are also among the numerous compounds identified from the seeds of *P. speciosa* [117,119,120,124]. The percentage composition of **60**, **61**, **62** and a triterpenoid **49** in the plant was reported as 3.42%, 2.18%, 2.29%, and 0.71% *w/w*, respectively [37]. In the case of *P. biglobosa*, the percentage composition of **60**, **61** and **62** in the seeds is higher with values of 55.7%, 3.42%, 37.1% for the unfermented, and 56.8%, 3.38%, 35.9% for the fermented, respectively, indicating that fermentation process may lower **61** and **62**, but increases **60** contents [129]. Meanwhile, Akintayo (2004) had recorded **60** as the most abundant compound in *P. biglobosa* seeds, constituting approximately 39.5% *w*/*w*. Compound **60** was isolated as a pure compound through column chromatographic separation of benzene fraction of *P. bicolor* leaves [42].

### 4.3. Miscellaneous Compounds

**Figure 5:**
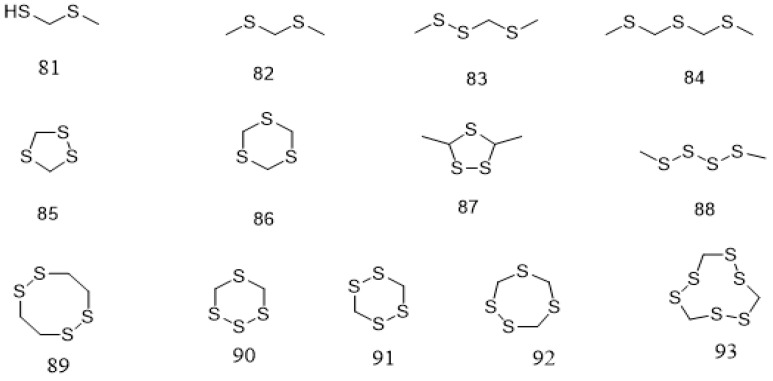
Structural formulas of cyclic polysulfides **81**–**93**, as previously listed in Table 2.

In addition to polyphenolic and terpenoids, several other compounds that are mainly volatile including aldehydes, esters, pyrazines, ketones, fatty acids, benzenes, alcohols, amines, sulfides, alkanes, and alkenes have been reported from *Parkia* species (Table 2). These compounds are identified mainly from the seeds. Compound **81** is identified from the natural product for the first time in pentane/dichloromethane fraction of *P. speciosa* seed using GC/ToF-MS [125]. A greater number of these compounds is identified through phytochemical quantification using different spectroscopic methods. Seven constituents are detected from the fresh seeds of *P. speciosa* through GC/ToF/MS and the compounds are dominated by linear polysulfide, alcohol, and 3′-thiobis-didodecyl ester. Other major compounds include palmitic acid, arachidonic acid, linoleic acid, linoleic acid chloride, and myristic acid [124]. However, cyclic polysulfides are the major constituents found in cooked *P. speciosa* seeds (Figure 5) [125]. In addition, some minor components, such as **82**–**84** are also identified. Meanwhile, **132** content in *P. speciosa* seed was reported to be 4.15 mg/100 g [37], but that of *P. biglobosa* in a recent study was found to be much higher (53.47 mg/100 g). Phospholipid content of *P. biglobosa* seeds was about 451 mg/100 g [122]. The seeds also contain palmitic acid, stearic acid, oleic acid, arachidic acid, and linoleic acid, the most abundant fatty acid [22,121,130]. Similar fatty acids are also reported in the raw seeds of *P. roxburghii* chloroform/methanol extract, in addition to total free phenol (0.56 g/100 g seed flour) and tannins (0.26 g/100 g seed flour) contents [41].

## 5. Pharmacological Activities of *Parkia* Species

Numerous bioactive constituents such as phenolics, flavonoids, terpenoids, and volatile compounds present in *Parkia* species may account for its various health benefits, and therefore responsible for the vast pharmacological properties (Table 3). However, only few species have been extensively studied.

### 5.1. Antimicrobial Activity

Various parts of many species of *Parkia* have good antimicrobial activities. They are most active against *S. aureus* and *E. coli* (Table 3). So far, there is still no clinical study conducted on the plants investigating the activity. Enormous reports have been made on antimicrobial activity of different parts of *P. biglobosa* such as leaves [23,65,131,132,133,134,135], stem barks [31,43,45,65,67,131,134,135,136,137], seeds [138], roots [34,38,139] and pods [133,140]. Furthermore, the stem barks and leaves of *P. clappertoniana* aqueous and methanol extracts investigated on some Gram-positive and Gram-negative bacteria revealed that both stem barks and leaves were effective in all tested organisms, but methanol extract was more potent [71]. The ethanol extract of both leaves and barks demonstrated growth inhibitory effects on multi-drug resistant *Salmonella* and *Shigella* isolates [73]. The ethanol extract of *P. platycephala* seeds tested against six bacteria strains and three yeasts showed no antimicrobial activity [141]. However, lectin obtained from the seed was reported to significantly enhance antibiotic activity of gentamicin against *S. aureus* and *E. coli* multi-resistant strains due to interaction between carbohydrate-binding site of the lectin and the antibiotic [142].

In *P. speciosa*, the water suspension of the seeds displays some remarkable inhibitory activity against bacteria isolated from the moribund fishes and shrimps—*S. aureus*, *A. hydrophila*, *S. agalactiae*, *S. anginosus* and *V. parahaemolyticus*—but no detectable activity against *E. coli*, *V. alginolyticus*, *E. tarda*, *C. freundii*, and *V. vulnificus* [143]. The methanol, chloroform, and petroleum ether extracts of the seeds demonstrate growth inhibitory effect against *H. pylori* [144], while the ethyl acetate extract against *E. coli*, but no effect on *S. typhi*, *S. sonnei*, and *S. typhimurium* [144]. The antimicrobial activity of *P. speciosa* is attributable to the presence of cyclic polysulfides **85** and **92**–**94** in the seeds [126]. However, possible mechanism of the polysulfides was not elucidated. Both pod extract and its synthesized silver nanoparticles exhibit antibacterial activity, with the latter shows higher activity against *P. aeruginosa* [145]. A similar antibacterial activity is also seen with aqueous extract of *P. speciosa* leaves and its silver nanoparticles against *S. aureus*, *B. subtilis*, *E. coli*, and *P. aeruginosa* [146]. Its bark methanol extract inhibits the growth of *Gloeophyllum trabeum*, but not *Pycnoporus sanguineus*, which effect is not seen with both sapwood and heartwood of the plant [147]. Its ethyl acetate extract of the peel also shows four times higher activity against *S. aureus* and three times higher against *E. coli* than streptomycin, but n-hexane extract exhibits lower activity [148].

Various parts of *P. timoriana* inhibit growth of *B. cereus*, *V. cholerae*, *E. coli*, and *S. aureus* [149]. Its leaf extract exhibits significant growth inhibitory effect against *E. coli*, *V. cholerae*, *S. aureus*, and *B. cereus* [150], while its gold and silver nanoparticles from dried leaves inhibit *S. aureus* growth. The activity is believed to be attributable to the accumulation and absorption of the gold and silver nanoparticles into *S. aureus* cell wall [151]. The methanol extract and semi-polar fractions (chloroform and ethyl acetate) of the bark demonstrate significant inhibitory effects against *Neisseria gonorrhoeae*. The chloroform extract shows the best activity [97]. The aqueous extract of the seed, leaf and skin pod also possess antimicrobial activity [152].

Acetone, ethanol and aqueous extracts of *P. biglandulosa* stem bark were among the plant extracts that show the highest antimicrobial activity against bacteria and fungi [69] as well as plant pathogenic bacteria [153]. The methanol extract of the leaves also shows remarkable growth inhibition against *E. coli*, *P. aeruginosa*, and *S. aureus* [154]. Investigation on *P. bicolor* indicates that ethyl acetate, ethanol, and aqueous extracts of the leaves demonstrate a concentration-dependent growth inhibitory effect against some Gram-positive of bacteria such as *E. coli*, *S. aureus*, *P. aeruginosa*, *A. niger*, *B. cereus*, and a fungus, *C. utilis* [23]. Methanol, ethyl acetate, and water extracts of the root also exhibit different degrees of inhibition against some common human pathogenic bacteria including *C. diphtheriae*, *K. pneumoniae*, *P. mirabilis*, *S. typhi*, and *S. pyogenes* [28]. The possible mechanism of the antimicrobial activity of *Parkia* plants are yet to be determined. However, terpenoids from the plants could induce lipid flippase activity in the bacterial cellular membrane which then enhances membrane damage for a better cell penetration [155]. Other possible actions could be by damaging bacterial protein, inhibiting DNA gyrase and DNA synthesis, which are yet to be confirmed in further studies.

Collectively, it can be concluded that the antimicrobial properties of *Parkia* plants depend on the species and parts of *Parkia* as well as solvent (polar and non-polar). Most of the published report are in in vitro evaluations, which do not assure the same outcomes in animal models and clinical setting. In the rise of resistant pathogenic bacteria to antimicrobial therapy, it is an urgent need to develop new antimicrobial agents, and phytoconstituents from plants like *Parkia* could be good candidates.

### 5.2. Antidiabetic Activity

*P. speciosa* is the most studied among other species for antidiabetic activity. Six studies comprising three in vitro and three in vivo studies have demonstrated hypoglycemic activity of the plant, but no clinical study has been conducted. Most of the studies have studied the activity in the seeds and pods [120,156,157,158].

Pericarps from *P. speciosa* show significant inhibitory activity (IC_50_ 0.0581 mg/mL; 89.46%) against α-glucosidase [156], an enzyme responsible for breaking down starch and polysaccharide into glucose [159]. The seeds also show inhibitory activity but at lower percentage (45.72%) [156]. In another study, the ethanol extract of the rind had the highest α-glucosidase inhibitory activity followed by the leaf and seed with IC_50_ of 4596 ppm, 54,341 ppm, and 67,425 ppm, respectively as compared with acarbose having 162,508 ppm [158].

An in vivo study conducted on both seeds and pods of *P. speciosa* in alloxan-induced diabetic rats, indicated that only chloroform extract of both pods and seeds exhibited strong glucose-lowering activity. The hypoglycemic activity of the seeds was higher than that of the pods (57% and 36%, respectively) [157]. A mixture of 66% β-sitosterol **60** and 34% stigmasterol **61** is believed to be responsible for the hypoglycemic effect of the seeds—demonstrated 83% decrease in blood glucose level (100 mg/kg body weight) compared to glibenclamide (111% at 5 mg/kg bw) [120]. Similarly, stigmast-4-en-3-one **63** was identified as the compound responsible for the 84% reduction in blood glucose level at 100 mg/kg bw of the pod extract of *P. speciosa* [123]. Both compounds (β-sitosterol and stigmasterol) are believed to reduce blood glucose level by regenerating remnant β-cells and stimulating insulin release [160] via augmentation of GLUT4 glucose transporter expression [161]. Stigmasterol is also reported to inhibit the ß-cells apoptosis [162].

In other *Parkia* species, methanol crude extracts and fractions of *P. timoriana* pods showed significant α-glucosidase and α-amylase inhibitory activities in streptozotocin-induced diabetic rats. Ethyl acetate fraction had the highest α-glucosidase inhibitory and moderate α-amylase inhibitory activities, with maximal reduction in blood glucose level back to normal observed on day 14 at the dose of 100 mg/kg body weight [18]. α-Amylase functions to hydrolyze starch into maltose and glucose [163]. Bioassay-guided chemical investigation of the most active ethyl acetate fraction revealed epigallocatechin gallate **4** and apigenin **14** were responsible for the antidiabetic activity [18].

Oral administration of *P. biglobosa* methanol and aqueous extracts of fermented seeds exhibited different degrees of hypoglycemic effects on fasting plasma glucose when tested on alloxan-induced diabetic rats after four weeks [164,165]. Oral administration of *P. biglobosa* seeds methanol extract (1 g/kg body weight) lowered blood glucose level by 44.1% at 8 h as compared with glibenclamide (37.9%) in alloxan-induced diabetic rats. Its chloroform fraction exerted maximum glucose-lowering effect (65.7%), while n-hexane fraction had the lowest (4.7%) [39]. As previously mentioned, similar underlying mechanism of the hypoglycemic activity of the plant species is suggested which is via an improvement in pancreatic islet functions to release insulin, while abolishing insulin resistance [166]. For future study directions, investigations on the effects of the plant extracts and pure compounds on insulin release and signaling pathways that might be involved in the glucose-lowering properties could be conducted. The compounds should also be studied clinically.

### 5.3. Anticancer Activity

Cancer is one of the diseases that cause death of millions worldwide. Dietary intake of raw seeds was also reported to significantly lower the occurrence of esophageal cancer in southern Thailand [167]. The methanol extract of *P. speciosa* seeds exhibited a moderate antimutagenic activity in the Ames test [168], but weak activity in Epstein–Barr virus inhibitory assay [169]. The methanol extract of the seed coats demonstrated selective cytotoxicity against MCG-7 and T47D (breast cancer), HCT-116 (colon cancer) and HepG2 (hepatocarcinoma) cells, while its ethyl acetate fraction only showed selective cytotoxicity against MCF-7, breast cancer cells [170].

Substances that enhance mitogenesis of lymphocytes may be useful as antitumor or antiproliferative and immunomodulator agents [171]. Lectin obtained from the *P. speciosa* seeds exerted mitogenic activity in both rat thymocytes and human lymphocytes by stimulating the incorporation of thymidine into DNA cell, which activity was comparable to the known T-cell mitogens like pokeweed mitogen, concanavalin A and phytohemagglutinin [6,172]. Lectins isolated from the seeds of *P. biglandulosa* and *P. roxburghii* have demonstrated antiproliferative effect on murine macrophage cancer cell lines—P 388DI and J774. The seed extract *P. roxburghii* also inhibits the proliferation of B-cell hybridoma cell line, HB98 [173], and HepG2 cells without affecting the normal cells [44]. The monosaccharide saponins **52**–**55** isolated from *P. bicolor* root also exhibit moderate antiproliferative effect IC_50_ ranging from 48.49 to 81.66 µM [118]. To our knowledge, the anticancer effects of *Parkia* extracts were only investigated in cell lines—limited to cell growth inhibition—not yet studied in in vivo models.

An in vitro study on human cancer cell lines has shown that the methanol extracts of *P. biglobosa* and *P. filicoidea* exhibit different degrees of antiproliferative activities on T-549 and BT-20 (prostate cancer), PC-3 (acute T cell leukemia Jurka), and SW-480 (colon cancer) at concentrations of 20 and 200 µg/mL. *P. biglobosa* also exhibits higher cytotoxic activity against all types of cancer cell lines used compared with *P. filicoidea* [174]. The antitumor property could be attributable to the antiangiogenic activity of some species of *Parkia* such as *P. biglandulosa* and *P. speciosa* extracts [170,175]. Angiogenesis or neovascularization is involved in metastasis of solid tumors. Methanol extract of the *P. speciosa* fresh pods was reported to exhibit antiangiogenic activity by more than 50% inhibition of microvessel outgrowth in rat aortae and human umbilical vein endothelial cells forming capillary-like structures in Matrigel matrix. The effect may be attributable to the ability of the compounds in the extract to form vacuoles in the cells [170], which is essential in maintaining the viability of the cells, therefore beneficial in the treatment of cancer owing to its capacity to prevent tumor neovascularization [176].

The plant bioactive compounds could also possibly increase apoptotic signaling pathway by elevating caspase activation as similarly shown by the same compounds in other plant species [177], as well as a direct inhibition on DNA synthesis, related to the ability to inhibit the expressions of several tumor- and angiogenesis-associated genes. Future studies should explore on the possible mechanism of action that are responsible for the anticancer activity. Additionally, future research on human studies is needed to confirm the outcomes seen in the laboratories.

### 5.4. Antihypertensive Activity

Antihypertensive activity of *P. biglobosa* seeds has been demonstrated in both animals and human. Only a clinical study was conducted which observed lower blood pressure, blood glucose and heart rate, high level of magnesium as well as improved lipid profile in patients with hypertension consuming fermented seeds of *P. biglobosa* in comparison with the non-consumption group [178]. Administration of 1.9 mg/mL of seed extract of *P. biglobosa* lowers the arterial blood pressure level in a rat model, possibly due to its ability to slow down the heart rate [179] and to induce vascular relaxation [180]. The latter effect is also seen with roasted seeds of the plant [180]. Other than the seeds, *P. biglobosa* stem bark aqueous extract also demonstrates good hypotensive effect in adrenaline-induced hypertensive female rabbits, which effect is comparable to antihypertensive drugs, propranolol and nifedipine [181]. The hypotensive properties of *P. biglobosa* could be owing to its main phytochemicals−phenolics and flavonoids. Catechin and its derivatives are among the most common compounds detected in the plant. These compounds promote vasorelaxation [182] by modulating nitric oxide availability [183] and inhibiting angiotensin-converting enzyme (ACE) [184], in addition to a reduction in oxidative stress [185], leading to blood pressure-lowering effects of the plant extract. The fermented seeds also decrease plasma triglyceride and cholesterol levels in Tyloxapol-induced hyperlipidemic rats [186], and platelet aggregation [187].

*P. speciosa* empty pod extract has been reported to prevent the development of hypertension in rats given L-NG-nitroarginine methyl ester (L-NAME), a nitric oxide synthase inhibitor, possibly due to its ability to prevent nitric oxide loss [122], which is dependent on the availability of endothelial nitric oxide synthase [188], as well as to inhibit ACE and oxidative stress and inflammation [112]. Both oxidative stress and inflammation are known to play important roles in the pathogenesis of hypertension (Siti et al. 2015). Active peptide obtained from hydrolyzed *P. speciosa* seeds displays ACE-inhibitory effect, ranging from 50.6% to 80.2%, which effect is not observed in the non-hydrolyzed seeds, possibly due its long and bulky structure [189,190]. However, the study of Khalid and Babji (2018) has demonstrated that the aqueous extract of the seeds also possesses ACE inhibitory activity [191]. These studies suggest that the blood pressure-lowering effect of the *P. speciosa* is most likely due to its ACE inhibitory property and nitric oxide regulation, attributable to its rich contents of polyphenols and the presence of peptide. Future studies should involve isolation of the active compounds which have a potential to be developed as a specific inhibitor of ACE. Other possible mechanisms—specific receptor antagonism, such as adrenoceptors and calcium channels, or modification of signaling pathways—of blood pressure-lowering effects of the plant extract or compounds should be explored further.

### 5.5. Antidiarrheal Activity

The antidiarrheal effect of *Parkia* plants has been investigated using many models such as castor oil- and magnesium-induced diarrhea. The aqueous extract of *P. filicoidea* stem barks reduces the frequency of stooling in rats with castor oil-induced diarrhea, comparable with loperamide [192]. The aqueous and ethanol extract of *P. biglobosa* leaves and stem barks also exhibit similar antidiarrheal activity to loperamide, seen as a reduction in stooling frequency and intestinal volume [45,59,193]. These effects could be attributable to its inhibitory capacity on the propulsive movement of gastrointestinal tract smooth muscles [45]. Medicinal plants are believed to exert antidiarrheal activity by enhancing the opening of intestinal potassium channel and stimulating Na^+^/K^+^-ATPase activity, as well as decreasing intracellular calcium concentration, which then promotes gastrointestinal smooth muscle relaxation, leading to diminished diarrhea [194,195,196]. The potential of these plants as agents to reduce diarrhea can be explored further in irritable bowel syndrome or chemotherapy-induced diarrhea. Their effects on intestinal mucosal barrier, tight junction proteins and inflammatory cytokines among others can be examined.

### 5.6. Antiulcer Activity

The gastroprotective effect of *Parkia* plants was seen in three species which were *P. speciosa*, *P. platycephala* and *P. biglandulosa* (Table 3). The leaves and seeds of *P. speciosa* protected against ethanol- and indomethacin-induced gastric ulcer in rats, observed by reductions in the gastric ulcer index and acidity of gastric juice [197,198]. Lesser collagen and fibrotic ulcer were significantly diminished in the extract-treated group [197]. The ethanol extract of *P. platycephala* also showed protective effect in gastric mucosal injury models induced by ethanol, ischemia-reperfusion, and ethanol-HCl. However, the extract could not protect against indomethacin-induced gastric lesion [199]. These plants are rich in flavonoids. The compounds like catechin and quercetin confer antiulcer effects possibly by eradicating the formation of ROS and modulating mucin metabolism in the gastrointestinal tract [200,201,202]. Other possible protective mechanisms could be by reducing gastric acid secretion, thereby decreasing gastric acid pH, as seen with cinnamic caffeic, p-coumaric or ferulic acids—the compounds that are present in the plants [203]. Studies on other possible effects of the extracts or bioactive components such as proton pump inhibition could be of interest. In future, the compounds that are responsible for the protective effects should be identified and the possible protective signaling mechanisms should be elucidated. Moreover, clinical trials can be performed to assess the potential use of *Parkia* extracts as an antiulcer agent.

### 5.7. Antianemic Activity

The fermented seeds of *P. biglobosa* are a rich source of essential minerals such as iron, calcium, thiamine, and phosphorus [57] which are necessary in forestalling either iron or non-iron deficiency anemia. Therefore, the antianemic capacity of *P. biglobosa* could be owing to its nutritional composition. The fermented seeds of *P. biglobosa* in combination with other fermented products were reported to be beneficial in the management of anemia as it increased hemoglobin, red blood cells, white blood cells, and packed cell volume [204]. The ethanol extract of *P. speciosa* seeds were also investigated in NaNO_2_-induced anemic mice. At doses of 400 and 700 mg/kg, an elevation of hemoglobin levels was noted to 0.92 and 0.82 g/dL, respectively [205]. The exact mechanism of how *P. speciosa* acts to decrease anemia is still unclear. It could be due to its rich source of the minerals, particularly the iron [171]. Another possible mechanism would be stimulation of erythropoiesis process. Both extracts of *P. biglobosa P. biglobosa* and *P. speciosa* can be developed as an alternative iron supplement. However, the effectiveness should be evaluated clinically.

### 5.8. Anti-Inflammatory Activity

Inflammatory reaction is involved in almost all clinical manifestation. Hence, anti-inflammatory activity of certain plant extracts could be of benefit. Anti-inflammatory activity of *P. biglobosa* stalk [206], seeds and stem bark [29], *P. speciosa* pods [187,188] and seeds [84], as well as *P. platycephala* seeds [207] have been reported using various models of inflammation.

The protective effects of *P. biglobosa* is believed via its inhibitions on the lipoxygenase and cyclooxygenase pathways [206], leading to inhibition of pro-inflammatory cytokine release and stimulation of anti-inflammatory cytokine [208], as well as increment on membrane stabilization [209]. While the *P. speciosa* exerts its anti-inflammatory by downregulating nuclear factor kappa B cell (NF-κB) and p38 mitogen-activated protein kinase (MAPK) pathways [187,188]. It is obvious that the plant bioactive components attenuate inflammation by regulating inflammatory and MAPK signaling pathways, which could lead to reduced formation of inflammatory mediators such as cytokines. To date, no study has identified the anti-inflammatory compounds from *Parkia*, which warrants further studies on this aspect, either in experimental animals or human studies.

### 5.9. Antioxidant Activity

Polyphenolic compounds present in plant foods have been reported to be responsible for their antioxidant activity due to their ability to serve as a hydrogen donor and reducing agent (Amorati and Valgimigli 2012). Both fermented and unfermented seeds of *P. biglobosa* have been reported to contain an appreciable amount of phenolic contents [210,211]. *P. timoriana* pods are also rich in total phenolic and flavonoid contents [212]. The antioxidant capacity of the leaves and seeds of *P. speciosa* has been reported to be relatively lower than that of the empty pods and seed mixture, suggesting that the pods possess higher antioxidant contents than other parts of the plant [37,176]. The difference in geographical location may affect the composition of the antioxidant compounds in plants. It was reported that *P. speciosa* seeds collected from central Peninsular Malaysia had higher antioxidant capacity than the southern and southwestern regions [213]. The compounds present in the plants attenuate oxidative stress possibly by activating Nrf2/Keap1 and MAPK signaling pathways, leading to enhanced expressions of Nrf2 and antioxidant enzymes, such as heme oxygenase-1 [214]. *P. speciosa* extracts of seed coats and pods could also reduce the risk of hemolysis by inhibiting Heinz body production in the erythrocytes incubated with a hemolytic agent [215], indicating the ability of the extracts to inhibit oxidative destruction of erythrocyte. The finding suggests a potential of the plant extract to reduce hemolytic jaundice, which warrants further research.

### 5.10. Other Pharmacological Activities

Other than previously mentioned activities, the *P. biglobosa* extract has also been demonstrated to have antimalarial effect [11], whereas *P. clappertoniana* [75] and *P. biglobosa* [216] show nephro- and hepatoprotective effects, respectively (Table 3). *P. pendula* seeds also enhance wound healing in immunosuppressed mice [217]. However, extensive studies regarding these effects were not performed. Further studies need to be conducted to explore the possible mechanisms that are involved in the aforementioned beneficial effects.

## 6. Toxicity

Daily consumption of cooked pods of *P. roxburghii* does not impose any significant adverse effect [218]. However, eating raw pods may result in bad breath owing to its rich content in volatile disulfide compounds, which are exhaled in breath and the odor can persist for several hours (Meyer, 1987). Many substances have been identified or isolated from *Parkia* seed, such as lectins, non-protein amino acids, and alkaloids [219]. However, no acute mortality and observable behavioral change were recorded at doses up to 2000 mg/kg ethyl acetate fraction of *P. roxburghii* pod in rats [18]. Investigation on acute and sub-acute toxicity profiles of the aqueous and ethanol extracts of the stem bark of *P. biglobosa* showed that the oral median lethal dose (LD_50_) was higher than 5000 mg/kg for both extracts in rats [36]. However, in another report, LD_50_ values of the leaves, stems and roots in an acute toxicity study were within the range of 500–5000 mg/kg body weight of fish, suggesting that they are only slightly toxic and, therefore, not potentially dangerous. The adverse effects included respiratory distress and agitated behavior [220]. Apart from the barks of *P. biglobosa*, the pods also possess the piscicidal activity that can be used in the management and control of fishponds to eliminate predators [220,221]. Fatty acids and oils identified from the seeds of *P. biglobosa* and *P. bicolor* were reported to be non-toxic [22].

The aqueous extract of *P. clappertoniana* seeds showed no observable maternal and developmental toxicity at 100–500 mg/kg when given orally to Sprague-Dawley rats and mice at different gestational age ([17]. *P. platycephala* leaves at 1000 mg/kg on the other hand, caused decreases in body mass, food and water consumption in rats. It also shortened the proestrus and prolonged diestrus phases, as well as reduced uterine weight, suggestive of possible alterations on hormonal levels, but no obvious toxicity on other organs [53]. Oral administration of the leaves of *P. speciosa* for 14 days showed no significant histopathological toxicity or mortality in rats at up to 5000 mg/kg [198]. In vitro, the plant pods (100 μg/mL) showed no significant cytotoxic effect on normal cell lines [170]. Consumption of the seeds up to 30 pieces in a serve does not produce any adverse effects [176].

## 7. Conclusions

Enormous reports demonstrate that plants from genus Parkia possess medicinal values, attributable to the presence of pharmacological active compounds. Taken together, two most studied species, *P. biglobosa* and *P. speciosa*, show potential as antidiabetic, antihypertensive, and antimicrobial, to name a few. Phytochemical investigations indicated terpenoids (monoterpenoids, diterpenoids, and triterpenoids), phenolics acids and flavonoids (flavonols, isoflavone, flavanone, and flavan-3-ols) are the major chemical constituents present in the species of this genus, which are responsible for their diverse pharmacological activities. It seems that certain phytoconstituents in *Parkia* have their unique pharmacological effects. β-Sitosterol and stigmasterol, for instance, could be investigated further and be developed as hypoglycemic agents; cyclic polysulfides, such as antimicrobials; lectins and monosaccharide saponins for anticancer treatment; and polyphenols, most possibly catechin and its derivatives, and active peptides for blood pressure-lowering effect. The pharmacological properties studied in vitro and in vivo of these compounds should be confirmed in clinical studies. In order to carry out this, there is a need to develop a method, which is effective and cheap to isolate the bioactive constituents in bulks. The potential toxicity and safety of the compounds, as well as their possible protective mechanisms, should also be determined before administration into humans. However, research on other bioactive compounds should continue. It is hoped that discoveries of novel agents from these plants could provide an alternative to the current modern medicine.

## Figures and Tables

**Table 1:** The medicinal uses of plants from genus *Parkia*.

Species	Part Used	Method of Preparation	Medicinal Uses	Region/Country	Reference
*P. bicolor*	Stem bark	Pulverized powder	Wound healing	West coast of Africa and Nigeria	[23]
	Tree		Diarrhea, dysentery	Southwest Nigeria	[55]
	Stem barks	Decoction	Bad cough, measles, and woman infertility	Cameroon	[28]
	Stem barks	Decoction	Diarrhea and skin ulcers	Ghana	[56]
*P. biglobosa*	Roots & bark	Paste	Dental disorder	Ivory Coast	[29]
	Seed and stem bark	Fresh seeds	Fish poison	West Africa	[57,58]
	Root	Decoction combined with other plants	Infertility	Nigeria	[58]
		Bark infusion with lemon	Diarrhea	Nigeria	[59]
	Stem bark		Anti-snake venom	Nigeria	[60]
	Bark	Paste, decoction	Wound healing leprosy, hypertension, mouth wash, toothpaste	Nigeria	[22,23]
	Leaves and roots	Eyesore	Lotion	Gambia	[23]
	Bark	Hot decoction	Fever	Gambia	[23]
	Bark	Decoction	Malaria, diabetes, amenorrhea, and hypertension	Senegal, Mali, Ghana Togo, and South Africa	[11,40,61,62,63]
	Roots and bark	Decoction of the roots with *X*imenia *americana*	Weight loss	Burkina Faso	[64]
	Stem bark	Boiled bark	Diarrhea, conjunctivitis, severe cough, and leprosy	West Coast Africa	[23,65,66]
	Leaves	Decoction	Violent colic chest and muscular pain	Northern Nigeria	[38]
	bark	Infusion	Dental caries and astringent	Guinea Bissau	[67]
*P. biglandulosa*	Seed bark	Saponins	Astringent	India	[68]
	Stem bark		Hemagglutination, ulcer	India	[69]
	Tree		Inflammation and ulcer	India	[70]
*P. clappertoniana*	Tree		Hypertension	Southwest Nigeria	[55]
	Root		Dental caries and conjunctivitis	African	[71,72]
	Seed	Crudely pounded	Labor induction	Ghana	[17]
	Tree		Diarrhea	Kaduna and Nigeria	[73]
	Leaves and bark	Maceration	Epilepsy	Northern Nigeria	[74]
	Stem bark		Chickenpox and measles	Southwest Nigeria	[24]
	Tree		Diabetes, leprosy, and ulcers	Ghana	[75]
	Tree		Mouthwash and toothache	Nigeria	[76]
	Tree		Eczema and skin diseases	Nigeria	[77]
	Bark	Infusion	Hernia	Ghana	[75]
*P. pendula*	Leaves bark		Genital bath	Netherland	[78]
	Bark	Decoction	Malaria	Brazil	[79]
*P. speciosa*	Seed	Eaten raw or cooked oral decoction	Diabetes	Malaysia	[80]
	Leaves	Pounded with rice and applied on the neck	Cough	Malaysia	[30]
	Root	Decoction	Skin problems	Southern Thailand	[21]
	Root	Decoction taken orally	Hypertension and diabetes	Malaysia	[26]
	Fruit	Eaten raw	Diabetes	Malaysia	[30]
	Seed	Eaten raw	Detoxification and hypertension	Singapore	[81]
			Ringworm	Malaysia	[82]
	Leaf	Decoction	Dermatitis	Indonesia	[20]
	Root	Oral decoction	Toothache	Malaysia	[27]
	Tree		Heart problem, constipation and edema	India	[83,84]
	Leaves		Dermatitis	Indonesia	[85]
	Seed		Loss of appetite	Indonesia	[86]
	Seed	Cooked	Kidney disorder	West Malaysia	[87]
*P. timoriana*	Bark and twig	Decoction of bark and twig paste	Diarrhea, dysentery, and wound	India	[88]
	Bark	Decoction used to bath	Fever	Gambia	[89]
	Pulp bark	Mixed with lemon	Ulcer and wound	Gambia	[89]
	Fruit		Diabetes	Thailand	[90]
	Pod	Pounded in water	Hair washing, skin diseases, and ulcers	India	[19]
	Bark and leaves		Head washing, skin diseases, and ulcers	India	[19]
	Bark	Decoction with *Centella. asiatica* and *Ficus glomerata*	Diabetes	India	[16]
*P. roxburghii*	Tree	Tender pod and bark taken orally	Diarrhea, dysentery, intestinal disorder, and bleeding piles	India	[91]
	The fruit or young shoot	Green portion of the fruit mixed with water to be taken orally	Dysentery, diarrhea, food poisoning, wound, and scabies	India	[92]
	Seed	Grounded and mixed with hot water	Postnatal care, diarrhea, edema and tonsillitis	Malaysia	[93]
	Pod		Diabetes, hypertension, and urinary tract infections	India	[18]
	Leaves, pod, peals, and bark		Diarrhea and dysentery	India	[94]
	Stem bark	Hot water extraction	Diarrhea and dysentery	India	[95]
	Bark	Turn into paste	Used as plaster for eczema	India	[25]
*P. javanica*	Bark, pod, and seed	Taking orally as vegetable	Dysentery and diarrhea	India	[15]
	Tree		Inflammation	India	[96]
	Bark fruit		Dysentery and piles	India	[51]
			Stomachache and cholera	India	[97]
	Bark and leaves	Lotion	Sores and skin diseases		[98]
	Tree		Diarrhea, cholera dysentery, and food poisoning	India	[99]

**Table 2:** Phytochemical compounds from *Parkia*.

Structure Number	Type	Compound	Species	Part	Reference
**Polyphenolics**					
**1**	Flavanol	Catechin	*P. speciosa*	Pod	[107]
			*P. biglobosa*	Root/bark	[106]
			*P. javanica*	Edible part	[108]
**2**	Flavanol	Epicatechin	*P. speciosa*	Pod	[107]
			*P. javanica*	Edible part	[108]
**3**	Flavanol	Epigallocatechin	*P. biglobosa*	Root/bark	[111]
			*P. javanica*	Edible part	[108]
**4**	Flavanol	Epigallocatechin gallate	*P. roxburghii*	Pod	[18]
			*P. biglobosa*	Root/bark	[106,111]
**5**	Flavanol	Epicatechin-3-*O*-gallate	*P. biglobosa*	Bark	[111]
**6**	Flavanol	4-*O*-methyl-epigallocate-chin	*P. biglobosa*	Bark	[111]
**7**	Flavanol	Epigallocatechin-*O*-glucuronide	*P. biglobosa*	Root/bark	[106]
**8**	Flavanol	Epicatechin-*O*-gallate-*O*-glucuronide	*P. biglobosa*	Root/bark	[106]
**9**	Flavanol	Epigallocatechin-*O*-gallate-*O*-glucuronide	*P. biglobosa*	Root/bark	[106]
**10**	Flavanol	Theaflavin gallate	*P. speciosa*	Pod	[112]
**11**	Flavonol	Kaempferol	*P. speciosa*	Pod	[107]
			*P. javanica*	Edible part	[108]
**12**	Flavonol	Quercetin	*P. speciosa*	Pod	[107]
**13**	Flavonol	Hyperin	*P. roxburghii*	Pod	[18]
**14**	Flavonol	Apigenin	*P. speciosa*	Pod	[112]
**15**	Flavone	3,7,3′,4′-Tetrahydroxyflavone	*P. clappertoniana*	Seeds	[113,114]
**16**	Flavone	7-Hydroxy-3, 8, 4′-trimethoxyflavone	*P. clappertoniana*	Leaves	[115]
**17**	Flavone	2′-Hydroxy-3,7,8,4′,5′′pentamethoxyflavone	*P. clappertoniana*	Leaves	[115]
**18**	Flavone	Nobiletin	*P. speciosa*	Pod	[112]
**19**	Flavone	Tangeritin	*P. speciosa*	Pod	[112]
**20**	Flavonol	Myricetin	*P. javanica*	Edible part	[108]
			*P. speciosa*	Pod	[112]
**21**	Flavonol glycoside	Rutin	*P. javanica*	Edible part	[108]
			*P. speciosa*	Pod	[112]
**22**	Flavonol glycoside	Didymin	*P. speciosa*	Pod	[112]
**23**	Methoxy flavonol	Isorhamnetin	*P. javanica*	Edible part	[108]
**24**	Flavone	Luteolin	*P. javanica*	Edible part	[108]
**25**	Flavanone	Naringenin	*P. javanica*	Edible part	[108]
**26**	Flavanone	Naringenin-1-4′-di-*O*-ß-d-glucopyranoside	*P. biglobosa*	Fruit pulp	[109]
**27**	Isoflavone	Genistein	*P. javanica*	Edible part	[108]
**28**	Isoflavone	Daidzein	*P. javanica*	Edible part	[108]
**29**	Phenolic acid	Gallic acid	*P. speciosa*	Pod	[107]
			*P. bicolor*	Root	[28]
**30**	Phenolic acid	Methyl gallate	*P. bicolor*	Root	[28]
**31**	Phenolic acid	Hydroxybenzoic acid	*P. speciosa*	Pod	[107]
**32**	Phenolic acid	Vanillic acid	*P. speciosa*	Pod	[107]
**33**	Phenolic acid	Chlorogenic acid	*P. speciosa*	Pod	[107]
			*P. javanica*	Edible part	[108]
**34**	Phenolic acid	Ellagic acid	*P. speciosa*	Pod	[107]
**35**	Phenolic acid	Punicalin	*P. speciosa*	Pod	[112]
**36**	Phenolic acid	Caffeic acid	*P. speciosa*	Pod	[107]
			*P. javanica*	Edible part	[108]
**37**	Phenolic acid	Cinnamic acid	*P. speciosa*	Pod	[107]
**38**	Phenolic acid	*P*-Coumaric acid	*P. speciosa*	Pod	[107]
			*P. javanica*	Edible part	[108]
**39**	Phenolic acid	Ferulic acid	*P. speciosa*	Pod	[107]
			*P. javanica*	Edible part	[108]
**40**	Phenolic acid	Coutaric acid	*P. speciosa*	Pod	[112]
**41**	Phenolic acid	Caftaric acid	*P. speciosa*	Pod	[112]
**42**	Phenolic	1-(*w*-Feruloyllignoceryl) -glycerol	*P. biglobosa*	Bark	[111]
**43**	Phenolic	1-(*w*-Isoferuloylalkanoyl) -glycerol	*P. biglobosa*	Bark	[111]
**44**	Phenolic	Malvidin	*P. speciosa*	Pod	[112]
**45**	Phenolic	Primulin	*P. speciosa*	Pod	[112]
**46**	Pheny propanoid	Parkinol	*P. javanica*	Leaves	[110]
**47**	Phenol	2-Methoxy phenol	*P. biglobosa*	Seed	[116]
**48**	Phenol	2,4-Disiopropyl-phenol	*P. biglobosa*	Seed	[116]
**Terpenoid and steroid**					
**49**	Triterpenoid	Lupeol	*P. biglobosa*	Bark	[111]
			*P. bicolor*	Root	[28]
			*P. speciosa*	Seeds	[117]
**50**	Monoterpenoid	Limonene	*P. biglobosa*	Seed	[116]
**51**	Triterpenoid	Ursolic acid	*P. javanica*	Leaf/stem	[42]
**52**	Triterpenoid	Parkibicoloroside A	*P. bicolor*	Root	[118]
**53**	Triterpenoid	Parkibicoloroside B	*P. bicolor*	Root	[118]
**54**	Triterpenoid	Parkibicoloroside C	*P. bicolor*	Root	[118]
**55**	Triterpenoid	Parkibicoloroside D	*P. bicolor*	Root	[118]
**56**	Triterpenoid	Parkibicoloroside E	*P. bicolor*	Root	[118]
**57**	Monoterpenoidal glucoside	8-*O*-p-Hydroxl-6′-*O*-p-coumaryl-missaeno-sidic acid	*P. javanica*	Leaf	[42]
**58**	Monoterpenoidal glucoside	7-*O*-E-3,4-Dimethoxycinnamoyl-6′-*O*-ß-d-glucopyranosylloganic acid	*P. javanica*	Leaf	[42]
**59**	Diterpene	16-*O*-Methyl-cass-13(15) ene-16,18-dionic acid	*P. bicolor*	Root	[118]
**60**	Steroid	β-Sitosterol	*P. speciosa*	Seed	[117,119,120]
			*P. javanica*	Leaf/stem	[42]
			*P. biglobosa*	Seed oil	[121,122]
**61**	Steroid	Stigmasterol	*P. speciosa*	Seed	[117,119,120]
			*P. biglobosa*	Seed oil	[121,122]
**62**	Steroid	Stigmasterol methyl ester	*P. speciosa*	Seed	[117,119]
**63**	Steroid	Stigmast-4-en-3-one	*P. speciosa*	Seed	[123]
**64**	Steroid	Stigmasta-5,24(28)-diene-3-ol	*P. speciosa*	Seed	[117]
**65**	Steroid	Campesterol	*P. speciosa*	Seed	[117,119]
			*P. biglobosa*	Seed oil	[121,122]
**66**	Steroid	Stigmastan-6,22-diien,3,6-dedihydo-	*P. speciosa*	Seed	[119]
**Miscellaneous Compounds**					
**67**	Fatty acid	Arachidonic acid	*P. speciosa*	Seed	[117,119]
			*P. bicolor*	Seed	[22]
			*P. biglobosa*	Seed	[22]
**68**	Fatty acid	Linoleic acid chloride	*P. speciosa*	Seed	[117,119]
**69**	Fatty acid	Linoleic acid	*P. speciosa*	Seed	[117,119]
			*P. biglobosa*	Seed	[22]
			*P. bicolor*	Seed	[22]
**70**	Fatty acid	Squalene	*P. speciosa*	Seed	[117,119]
**71**	Fatty acid	Lauric acid	*P. speciosa*	Seed	[117,124]
**72**	Fatty acid	Stearic acid	*P. speciosa*	Seed	[117,119,124]
			*P. biglobosa*	Seed	[22]
			*P. bicolor*	Seed	[22]
**73**	Fatty acid	Stearoic acid	*P. speciosa*	Seed	[124]
**74**	Fatty acid	Eicosanic acid	*P. speciosa*	Seed	[124]
**75**	Fatty acid	Oleic acid	*P. speciosa*	Seed	[117,119,124]
**76**	Fatty acid	Palmitic acid	*P. speciosa*	Seed	[117,119,124]
			*P. biglobosa*	Seed	[22]
			*P. bicolor*	Seed	[22]
**77**	Fatty acid	Myristic acid	*P. speciosa*	Seed	[117,119,124]
**78**	Fatty acid	Undecanoic acid	*P. speciosa*	Seed	[119,124]
**79**	Fatty acid	Stearolic acid	*P. speciosa*	Seed	[119]
**80**	Fatty acid	Hydnocarpic acid	*P. speciosa*	Seed	[124]
**81**	Cyclic polysulfide	1,3-dithiabutane	*P. speciosa*	Seed	[125]
**82**	Cyclic polysulfide	2,4- Dithiapentane	*P. speciosa*	Seed	[125]
**83**	Cyclic polysulfide	2,3,5-Trithiahexane	*P. speciosa*	Seed	[125]
**84**	Cyclic polysulfide	2,4,6-Trithiaheptane	*P. speciosa*	Seed	[125]
**85**	Cyclic polysulfide	1,2,4-Trithiolane	*P. biglobosa*	Seed	[116,126]
			*P. speciosa*	Seed	[126,127,128]
**86**	Cyclic polysulfide	1,3,5-Trithiane	*P. speciosa*	Seed	[128]
**87**	Cyclic polysulfide	3,5-Dimethyl-1,2,4-trithiolane	*P. speciosa*	Seed	[128]
**88**	Cyclic polysulfide	Dimethyl tetrasulfid	*P. speciosa*	Seed	[128]
**89**	Cyclic polysulfide	1,2,5,6-Tetrathio-cane	*P. speciosa*	Seed	[128]
**90**	Cyclic polysulfide	1,2,3,5-Tetrathiane	*P. speciosa*	Seed	[128]
**91**	Cyclic polysulfide	1,2,4,5-Tetrathiane	*P. speciosa*	Seed	[128]
**92**	Cyclic polysulfide	1,2,4,6-Tetrathie-pane	*P. speciosa*	Seed	[126,128]
**93**	Cyclic polysulfide	1,2,4,5,7,8- Hexathiolnane	*P. speciosa*	Seed	[126]
**94**	Cyclic poly-sulfide	Lenthionine	*P. speciosa*	Seed	[117,124,126,128]
**95**	Esters	*n*-Tetradecyl acetate	*P. speciosa*	Seed	[124]
**96**	Esters	Methyl linoleate	*P. speciosa*	Seed	[124]
**97**	Esters	Ethyl linoleate	*P. speciosa*	Seed	[117,124]
			*P. biglobosa*	Seed	[116]
**98**	Ester	Butyl palmitate	*P. speciosa*	Seed	[117]
**99**	Esters	Ethyl palmitate	*P. speciosa*	Seed	[124]
**100**	Esters	Methyl palmitate	*P. speciosa*	Seed	[124]
**101**	Esters	Methyl laurate	*P. speciosa*	Seed	[124]
**102**	Esters	Dodecyl acrylate	*P. speciosa*	Seed	[124]
**103**	Esters	Methyl hexadecanoate	*P. biglobosa*	Seed	[116]
**104**	Ester	Ethyl stearate	*P. speciosa*	Seed	[117,124]
**105**	Ester	Methyl octadecanoate	*P. biglobosa*	Seed	[116]
**106**	Ester	Butyl stearate	*P. speciosa*	Seed	[124]
**107**	Ester	Propanoic acid, 3,3′-thiobis-didodecyl ester	*P. speciosa*	Seed	[124]
**108**	Ester	Linoleaidic acid methyl ester	*P. speciosa*	Seed	[119]
**109**	Alcohol	2,6,10,14-Hexadecatetraen-1-ol	*P. speciosa*	Seed	[117]
**110**	Alcohol	1-Octen-3-ol	*P. biglobosa*	Seed	[116]
**111**	Alcohol	3-Ethyl-4-nonanol	*P. speciosa*	Seed	[117]
**112**	Alcohol	1-Tridecanol	*P. speciosa*	Seed	[117,124]
**113**	Acid	Eicosanoic acid	*P. speciosa*	Seed	[117]
**114**	Acid	16-*O*-Methyl-cass-13(15)ene-16,18-dionic acid	*P. bicolor*	Root	[118]
**115**	Acid	Elaidic acid	*P. speciosa*	Seed	[117,124]
**116**	Pyrazine	2,5-Dimethyl pyrazine	*P. biglobosa*	Seed	[116]
**117**	Pyrazine	Trimethyl pyrazine	*P. biglobosa*	Seed	[116]
**118**	Pyrazine	2-Ethyl-3,5-dimethyl pyrazine	*P. biglobosa*	Seed	[116]
**119**	Ketone	2-Nonade-canone	*P. speciosa*	Seed	[117,124]
**120**	Ketone	2-Pyrrolidi-none	*P. speciosa*	Seed	[117]
**121**	Ketone	Cyclodecanone	*P. speciosa*	Seed	[124]
**122**	Alkane	Cyclododecane	*P. biglobosa*	Seed	[116]
**123**	Alkane	Tetradecane	*P. speciosa*	Seed	[119]
**124**	Benzene glucoside	3,4,5-Trimethoxyphenyl-1-*O*-ß-d-glucopy-ranoside	*P. bicolor*	Root	[118]
**125**	Aldehyde	2-Decenal	*P. speciosa*	Seed	[117]
**126**	Aldehyde	Cyclo-decanone-2,4-decadienal	*P. speciosa*	Seed	[117]
**127**	Aldehyde	Pentanal	*P. biglobosa*	Seed	[116]
			*P. speciosa*	Seed	[125]
**128**	Aldehyde	3-Methylthio-propanal	*P. biglobosa*	Seed	[116]
**129**	Aldehyde	Tetradecanal	*P. speciosa*	Seed	[119,124]
**130**	Aldehyde	Pentadecanal	*P. speciosa*	Seed	[117,124]
**131**	Aldehyde	Hexadecanal	*P. speciosa*	Seed	[117,124]
**132**	Amine	Hexanamide	*P. speciosa*	Seed	[117]
**133**	Oil	Vitamin E	*P. speciosa*	Seed	[117,124]

**Table 3:** Pharmacological activities of *Parkia species* extracts and fractions.

Activity	Species	Part	Type of Extract/Compound	Key Findings	References
Antimicrobial	*P. biglobosa*	Leaf, stem bark, and root	Methanolic and aqueous	Active against *S. aureus*, *B. subtilis*, *E. coli*, *P. aeruginosa*.	[38]
	*P. biglobosa*	Root bark	Aqueous and methanol	Active against *E. coli*, *S. aureus*, *K. pneumoniae*, *P. aeruginosa*. Activity: Aqueous > methanol	[34]
	*P. biglobosa*	Leaves and pod	Aqueous and ethanol	Active against *S. aureus*, *E. aerogenes*, *S. typi*, *S. typhimurium*, *Shigella spp*., *E. coli*, and *P. aeruginosa* (bacteria), *Mucor spp*., and *Rhizopus spp*. (fungi)	[133]
	*P. biglobosa*	Bark and leaves	Hydro-alcohol and aqueous	Active against *E. coli*, *S. enterica*, and *S. dysenteriae*. Activity: hydroalcoholic > aqueous	[65]
	*P. speciosa*	Seeds	Water suspension	Active against *S. aureus*, *A. hydrophila*, *S. agalactiae*, *S. anginosus*, and *V. parahaemolyticus* isolated from moribund fishes and shrimps	[143]
	*P. speciosa*	Seed peel	Ethyl acetate (EA) Hexane Ethanol	EA: Four times higher than streptomycin against *S. aureus* and three times higher for *E. coli*. Hexane: 50% inhibitory ability of streptomycin for both bacteria. Ethanol: no inhibition	[148]
	*P. speciosa*	Pod extract and its silver	Aqueous	Pod: active against *P. aeruginosa* Silver particles: active against *P. aeruginosa*	[145]
	*P. speciosa*	Sapwood, heartwood, and bark	Methanol	Bark: Active against *G. trabeum*. Sapwood and heartwood: No effect	[147]
	*P. speciosa*	Seeds	Chloroform, petroleum ether, Aqueous and methanol	Active against *H. pylori* except aqueous extract. Activity: chloroform > methanol > petroleum ether	[222]
	*P. speciosa*	Seed	Methanol Ethyl acetate	Methanol: active against *H. pylori*. Ethyl acetate: active against *E. coli* Both: no effect on *S. typhimurium*, *S. typhi*, and *S sonnei*	[144]
	*P. javanica*	Stem bark	Methanol	Good inhibitory activity against *E. coli*, *S. aureus S. pyogenes* found in chronic wound	[223]
	*P. javanica*	Stem bark	Methanol	Active against four *Vibrio cholerae* strains	[224]
	*P. javanica*	Leaves	Gold and silver nanoparticles	Good inhibitory activity against *S. aureus*	[151]
	*P. javanica*	Bark	Methanol extract and semi-polar fractions (chloroform and ethyl acetate)	Active against *Neisseria gonorrhoeae*. Chloroform showed the best activity	[97]
	*P. javanica*	Seeds, leaves and skin pods	Aqueous	Active against *S. aureus*, A*. hydrophila*, and *S. typhimurium* Not active against *E. coli*	[152]
	*P. clappertoniana*	Leaves and barks	Ethanol	Active against *Salmonellae* and *Shigella*	[73]
	*P. clappertoniana*	Stem bark and leaves	Aqueous and methanol	Active against *S. aureus* and *P. aeruginosa*. Methanol extract was more potent	[71]
	*P. biglandulosa*	Leaf	Methanol	Active against *E. coli*, *P. aeruginosa*, and *S. aureus*	[154]
	*P. filicoidea*	Stem barks	Aqueous, acetone and ethanol	Active against *S. aureus*, *K. pneumoniae*, *P. aeruginosa*, *S. viridans and B. subtilis*. Not active against *E. coli*	[50]
	*P. bicolor*	Leaves	Ethyl acetate, ethanol and aqueous	Active against *E. coli*, *S. aureus*, *P. aeruginosa*, *A. niger*, *B. cereus* and a fungus, *C. utilis*	[23]
	*P. bicolor*	Roots	Methanol, ethyl acetate and Aqueous	Active against *C. diphtheria*, *K. pneumoniae*, *P. mirabilis*, *S. typhi*, and *S. pyogenes*	[28]
	*P. pendula*	Seeds	Lectin	Reduced cellular infectivity of human cytomegalovirus in human embryo lung (HEL) cells.	[225]
Hypoglycemic	*P. speciosa*	Seeds and pods	Chloroform	Strong glucose-lowering activity in alloxan-induced diabetic rats Activity: seeds > pod	[157]
	*P. speciosa*	Rind, leaves and seeds	Ethanol	Inhibited α-glucosidase activity in rat Activity: rind > leaf > seed	[158]
	*P. speciosa*	Seed	Chloroform	Reduced plasma glucose levels in alloxan-induced diabetic rats	[120]
	*P. biglobosa*	Fermented seeds	Methanol and aqueous	Reduced fasting plasma glucose in alloxan-induced diabetic rats	[160,161]
	*P. biglobosa*	Seeds	Protein	Significantly increased lipid peroxidation product levels in brain and testes of diabetic rats	[226]
	*P. biglobosa*	Seeds	Methanol and fractions (chloroform and n-hexane)	Showed glucose-lowering effect Activity: chloroform > methanol > n-hexane	[40]
	*P. javanica*	Fruits	Ethyl acetate fraction	Reduced blood glucose inhibited α-glucosidase and α-amylase in streptozotocin-induced diabetic rats	[18]
Antitumor/ Anticancer	*P javanica*	Fruits	Aqueous methanol	Increased apoptosis in sarcoma-180 cancer cell lines	[227]
	*P javanica*	Seeds	Methanol	Caused 50% death in HepG2 (liver cancer cell) but not cytotoxic to normal cells	[44]
	*P javanica*	Seeds	Lectin	Inhibited proliferation in cancerous cell lines; P388DI and J774, B-cell hybridoma and HB98 cell line	[173]
	*P. speciosa*	Seed coats	Methanol extract	Demonstrated selective cytotoxicity to MCG-7 and T47D (breast cancer), HCT-116 (colon cancer)	[228]
	*P. speciosa*	Pods	Methanolic ethyl acetate fraction	Showed selective cytotoxicity on breast cancer cells MCF-7	[170]
	*P. biglobosa*	Leaves and stem	Methanol	Antiproliferative effect in human cancer cells T-549, BT-20, and PC-3	[174]
	*P. filicoidea*	Leaves	Methanol	Antiproliferative effect in in human cancer cells T-549, BT-20, and PC-3	[174]
Antiproliferative and anti-mutagenic	*P. biglandulosa*	Seeds	Lectin	T cell mitogen and antiproliferative against P388DI and J774 cancer cell lines	[173]
Antihypertensive	*P. speciosa*	Seeds	Aqueous	Showed moderate ACE-inhibitory activity in in vitro	[191]
	*P. speciosa*	Seeds	Peptide	Inhibited angiotensin-converting enzyme (ACE) in rats. No effect observed in non-hydrolyzed samples	[189,190]
	*P. speciosa*	Pods	Methanol	Prevented the increases in blood pressure and angiotensin-converting enzyme (ACE) and restored nitric oxide in hypertensive rat model	[112]
	*P. biglobosa*	Stem bark	Aqueous	Induced hypotension in adrenaline-induced hypertensive rabbits	[181]
	*P. biglobosa*	Roasted and fermented seeds	Aqueous	Induced relaxation in rat aorta precontracted with phenylephrine in the presence or absence of endothelium.	[180]
	*P. biglobosa*	fermented seeds	Aqueous	Lower blood pressure, blood glucose, and heart rate, high level of magnesium as well as improved lipid profile in patients with hypertension	[178]
Antidiarrheal	*P. biglobosa*	Stem bark	Aqueous and fractions	The extract of stem bark exhibit dose-dependent antidiarrheal activity at different concentrations in albino rats with castor oil-induced diarrhea	[45]
	*P. biglobosa*	Leaves and stem bark	Aqueous and ethanol	Reduced frequency of stooling in castor-oil induced diarrhea in rats	[193]
	*P. biglobosa*	Stem-bark	70% Methanol	The extract exhibited 100% protections at 100 and 200 mg/kg bw in the diarrheal rats	[59]
	*P. filicoidea*	Stem bark	Aqueous	Reduced frequency of stooling and improved transit time at 100 and 200 mg/kg bw	[192]
Antiulcer	*P. speciosa*	Leaves	Ethanol	Reduced mucosal injury and increased in periodic acid-Schiff (PAS) staining induced by ethanol	[198]
	*P. speciosa*	Seed	Ethanol	Decreased gastric juice acidity, lesion length, collagen content and fibrosis in indomethacin-induced peptic ulcer in rats	[197]
	*P. platycephala*	Leaves	Ethanol	Reduced gastric mucosal lesion induced by ethanol, ischemia-reperfusion and ethanol-HCl	[199]
Antianemic	*P. biglobosa*	Combination of fermented seed with other fermented products	Aqueous	Increased hemoglobin, red blood cell, white blood cell levels and packed cell volume in albino rats	[204]
	*P. biglobosa*	Seeds	Ethanol	Increased hemoglobin levels in NaNO_2_-induced anemic mice	[205]
	*P. speciosa*	Seeds	Ethanol	Increased hemoglobin levels in NaNO_2_-induced anemic mice	[205]
Antiangiogenic	*P.biglandulosa*	Fruit and β-sitosterol	Ethanol	The extract and the isolated compound showed antiangiogenic activity on the caudal fin of adult zebrafish	[175]
	*P. speciosa*	Pods	Methanol and water sub-extract	Inhibited more than 50% micro vessel outgrowth in rat aortae and HUVECs	[170]
Antimalarial	*P. biglobosa*	Stem bark	Methanol and fractions	Showed antiplasmodial activity caused by *P. berghei* and *P. falciparum*	[11]
Nephroprotective	*P. clappertoniana*	Seed	Aqueous	Reduced serum creatinine, Na, urine proteins and leukocytes and kidney weight in gentamicin-induced renal damage in rats	[75]
Hepatoprotective	*P. biglobosa*	Stem barks	Methanol	Reduced serum alanine and aspartate transaminases, and alkaline phosphatase in paracetamol-induced hepatotoxicity rat model	[216]
Wound healing	*P. pendula*	Seeds	Lectin	Increased skin wound repair in immunosuppressed mice	[217]
Anti-inflammatory	*P. speciosa*	Pods	Ethyl acetate fraction	Reduced iNOS activity, COX-2, VCAM-1 and NF-κB expressions in cardiomyocytes exposed to tumor necrosis factor-α	[229]
	*P. speciosa*	Pods	Ethyl acetate fraction	Reduced iNOS activity, COX-2, VCAM-1 and NF-κB expressions in HUVECs exposed to tumor necrosis factor-α	[230]
	*P. biglobosa*	Stalk	Methanol	Inhibited croton pellet granuloma formation and carrageenin-induced rat paw edema	[206]
	*P. biglobosa*	Seeds	Lectin	Lectin showed anti-inflammatory effect by inhibition of pro-inflammatory cytokine release and stimulation of anti-inflammatory cytokine release on peritonitis induced model mice	[208]
	*P. biglobosa*	Stem bark	Hexane	Reduced carrageenan- and PMA-induced edema in mice	[29]
	*P. biglobosa*	Fruit	70% Methanol	Increased percentage protection of the human red blood cell membrane	[209]
	*P. platycephala*	Seeds	Lectin	Lectin showed antinociceptive effect in the mouse model of acetic acid-induced	[207]
Antioxidant	*P. javanica*	Leaves	Hexane, ethyl acetate, and methanol	Methanol extract showed the highest antioxidant potential activities (DPPH test) of about 85% and (FRAP test) of about 0.9 mM Fe (II)/g dry	[231]
	*P. javanica*	Leaves	Aqueous, ethanol and methanol	All the extracts exhibited good antioxidant activity. The aqueous extract showed the highest values of 47.42 and 26.6 mg of ascorbic acid equivalent/g in DPPH and FRAP tests, respectively	[232]
	*P. javanica*	Pods	Methanol and acetone	High content of total phenolic and flavonoid. Showed high reducing power and strong radical scavenging activity.	[212]
	*P. javanica*	Fruit	Methanol	Showed increased DPPH and ferric-reducing power activities concentration-dependently	[210]
	*P. speciosa*	Pod	Methanol	Increased DPPH scavenging activity	[233]
	*P. speciosa*	Pod	Ethyl acetate fraction	Reduced NOX4, SOD1, p38 MAPK protein expressions and ROS level	[230]
	*P. speciosa*	Pod	Aqueous and ethanolic	Increased DPPH and ABTS scavenging activities, reduced lipid peroxidation Activity: ethanol > aqueous	[107]
	*P. speciosa*	Seeds	Ethanol	Extract exhibited significant activity (DPPH and FRAP tests)	[213]
	*P. speciosa*	Seed coats and pods	Ethanol	Reduced Heinz body formation in erythrocytes incubated with acetyl phenylhydrazine. Activity: seed coat > pods >	[215]
	*P. speciosa*	Pods	Ethanol	Increased DPPH scavenging activity	[234]
	*P. biglobosa*	Fermented and unfermented seed	Aqueous	Fermented seed increased reduction of Fe^3+^ to Fe^2+^.	[211]
	*P. biglobosa*	Stem bark	Aqueous-methanolic	Mitigated ferric-induced lipid peroxidation in rat tissues and increased scavenging activities against DPPH and ABTS, ferric-reducing ability	[235]
	*P. biglobosa*	Fruit	Methanol and hydro-ethanol	Increased DPPH scavenging activity and reducing power.	[210]
	*P. biglobosa*	Fruit	Hydroethanolic and methanol	Increased scavenging activity against DPPH free radical Activity: methanol > hydroethanolic	[210]

Abbreviations: HUVECs, human umbilical vein endothelial cells; DPPH, 2,2-Diphenyl-1-picrylhydrazy; ABTS, 2,20-Azinobis (3-ethylbenzothiazoline-6-sulphonic acid) diammonium salt; FRAP, ferric reducing antioxidant power; iNOS, inducible nitric oxide synthase; PMA, phorbol myristate acetate; COX-2, cyclooxygenase-2; VCAM-1, vascular cell adhesion moelcule-1; NF-κB, nuclear factor kappa-B; ACE, angiotensin converting enzyme; HEL, human embryo lung; PAS, periodic acid-Schiff; bw, body weight.

## Data Availability

Data is contained within the article.
